# Prognostic Significance of Preoperative PET-CT SUVmax in Resected Non-Small Cell Lung Cancer: A Single-Center Retrospective Study

**DOI:** 10.3390/medicina62061004

**Published:** 2026-05-22

**Authors:** Alper Yaşar, Zeynep Yüksel Yaşar, Sedat Yıldırım, Akif Doğan, Tuğba Kaya, Miray Aydoğan, Tuğba Başoğlu, Deniz Işık, Hatice Odabaş, Nedim Turan

**Affiliations:** 1 Department of Medical Oncology, Darıca Farabi Training and Research Hospital, 41700 Kocaeli, Turkey; 2 Department of Medical Oncology, Kartal Dr. Lutfi Kirdar City Hospital, University of Health Sciences, 34865 Istanbul, Turkey; dr_zeynepyuksel@hotmail.com (Z.Y.Y.); rezansedat@hotmail.com (S.Y.); tugbakaya89@hotmail.com (T.K.); mirayaydogan1991@gmail.com (M.A.); basoglutugba@gmail.com (T.B.); dnz.1984@yahoo.com (D.I.); odabashatice@yahoo.com (H.O.); turan.nedim@hotmail.com (N.T.); 3 Department of Medical Oncology, Sancaktepe Sehit Prof. Dr. Ilhan Varank Training and Research Hospital, University of Health Sciences, 34785 Istanbul, Turkey; drakifd@gmail.com

**Keywords:** FDG PET-CT, non-small cell lung cancer, SUVmax, overall survival, disease-free survival

## Abstract

*Background and Objectives*: Positron emission tomography with 18F-FDG (PET-CT) provides a quantitative measure of tumor metabolic activity through the maximum standardized uptake value (SUVmax) of lung tumors—a measure of metabolic activity that may have prognostic value in non-small cell lung cancer (NSCLC). This study evaluated whether preoperative tumor SUVmax predicts outcomes in resected NSCLC. *Materials and Methods*: This single-center retrospective study included 209 consecutive patients with resected NSCLC who had preoperative FDG PET-CT. SUVmax of the primary tumor was recorded, and patients were stratified into low- and high-SUVmax groups to evaluate survival outcomes. *Results*: Median age was 62 years and 77% were male. Histologic subtypes were adenocarcinoma (44%), squamous carcinoma (43%), and others (13%), with stage I–III distribution of 39.7%, 33.5%, and 26.8%, respectively. SUVmax demonstrated moderate discrimination for mortality (AUC = 0.652), with an optimal cutoff of 11.14. Patients with SUVmax ≥ 11.14 had significantly worse OS and DFS. However, on multivariate analysis, SUVmax was not an independent predictor of outcomes, while extracapsular invasion (OS) and adjuvant chemotherapy (DFS) remained significant. *Conclusions*: In this cohort of resected NSCLC, high preoperative SUVmax (≥11.14) was associated with more advanced tumor stage and worse OS/DFS but was not an independent prognostic factor after accounting for other variables. Tumor invasiveness and use of adjuvant therapy were stronger outcome predictors. Preoperative SUVmax may help identify high-risk patients when considered alongside established clinicopathologic factors.

## 1. Introduction

Non-small cell lung cancer (NSCLC) is the most common type of lung cancer (approximately 80–85% of cases) and remains a leading cause of cancer mortality worldwide [1]. Complete surgical resection remains the cornerstone of curative-intent treatment for resectable NSCLC [1]. Nevertheless, outcomes after surgery are heterogeneous: a substantial proportion of patients experience recurrence despite apparently complete resection and guideline-concordant management [1]. This clinical variability highlights the ongoing need for preoperative, readily available biomarkers that can improve risk stratification beyond anatomic staging, support individualized surveillance strategies, and potentially refine selection for perioperative systemic therapy [1].

18F-fluorodeoxyglucose positron emission tomography (FDG PET-CT) is routinely used for initial staging of NSCLC and provides semiquantitative measures of tumor glucose metabolism [2]. The maximum standardized uptake value (SUVmax) of the primary tumor is simple to obtain, widely reported, and biologically plausible as a marker of aggressive tumor behavior [2]. Over the last two decades, multiple studies have examined whether higher preoperative primary tumor SUVmax identifies patients at increased risk of recurrence and death following curative-intent surgery [2]. In a frequently cited meta-analysis, Berghmans and colleagues reported that primary tumor SUV measurements have prognostic value for survival in NSCLC [3]. More recently, Liu et al. performed a meta-analysis focused specifically on surgical NSCLC, demonstrating that higher SUVmax—as well as volumetric metabolic metrics such as metabolic tumor volume (MTV) and total lesion glycolysis (TLG)—was associated with a significantly higher risk of recurrence and mortality, supporting the clinical relevance of FDG PET/CT-derived biomarkers in the postoperative setting [2].

Despite these encouraging data, the clinical translation of SUVmax into postoperative decision-making remains limited by several factors. Reported SUVmax thresholds vary widely across cohorts due to differences in patient mix (including pathologic stage distribution), scanner technology, reconstruction parameters, uptake time, and analytic choices, creating uncertainty about which cutoff, if any, should be applied in routine practice [2,3]. In addition, while many analyses show prognostic associations in unadjusted models, the degree to which SUVmax adds independent prognostic information beyond established clinicopathologic factors (e.g., nodal status, tumor size, histology, and adjuvant therapy) requires careful evaluation in well-characterized surgical cohorts [2,3]. An individual patient data-oriented approach has been advocated to better define the magnitude and robustness of SUVmax as a prognostic variable, underscoring the importance of rigorous modeling and validation [4]. Modern surgical series continue to suggest that SUVmax may independently stratify recurrence risk after resection, supporting renewed interest in integrating metabolic information into postoperative risk assessment [5].

A higher SUVmax might indicate more aggressive biology and poorer prognosis [2]. Indeed, meta-analyses have shown that elevated primary tumor SUVmax is associated with worse disease-free and overall survival in surgically treated NSCLC [2]. Several retrospective studies in early-stage disease have reported that high SUVmax predicts recurrence and death [2]. For example, Um et al. found that in stage I NSCLC, higher SUVmax was significantly associated with shorter disease-free survival, even after multivariate adjustment [6]. Similarly, Kocaman et al. reported that in stage I NSCLC, SUVmax > 5.2 independently predicted both recurrence-free and overall survival [7]. Chou et al. showed that among stage IA NSCLC patients, tumor SUVmax > 4 dramatically increased relapse risk (HR ≈ 9) compared to lower values [8].

Conversely, some studies found weaker or no independent associations. Tapias et al. (2024) reported that preoperative SUVmax was not an independent predictor of overall survival in clinical stage IA NSCLC after multivariable analysis [9]. Thus, the prognostic impact of SUVmax in resected NSCLC remains controversial and may depend on patient selection and cutoff values [9].

Although prior studies and meta-analyses suggest that higher preoperative primary tumor SUVmax is associated with worse survival in surgically treated NSCLC, its independent prognostic value remains uncertain because reported cutoffs vary substantially across studies and the observed associations may be confounded by established clinicopathologic risk factors. Therefore, the key unresolved issue is whether SUVmax provides incremental prognostic information beyond standard pathological features in resected NSCLC. In this study, we aimed to evaluate the prognostic significance of preoperative primary tumor SUVmax in patients undergoing curative-intent resection for NSCLC and to determine whether SUVmax adds meaningful risk stratification beyond conventional clinicopathologic variables. By directly evaluating the incremental prognostic value of SUVmax in the context of established pathological risk factors, this study helps to clarify whether SUVmax provides independent clinical information or primarily reflects underlying aggressive tumor biology, thereby better defining its role in preoperative risk stratification.

## 2. Materials and Methods

This single-center retrospective cohort study included consecutive patients with histologically confirmed NSCLC who underwent curative-intent surgical resection between 2016 and 2023 and had available preoperative 18F-FDG PET/CT imaging. Patients were identified from the institutional lung cancer database according to prespecified inclusion and exclusion criteria. The study was conducted in accordance with the Declaration of Helsinki and was approved by the local institutional ethics committee; the requirement for individual informed consent was waived because of the retrospective, non-interventional design and anonymized data analysis.

**Figure 1:**
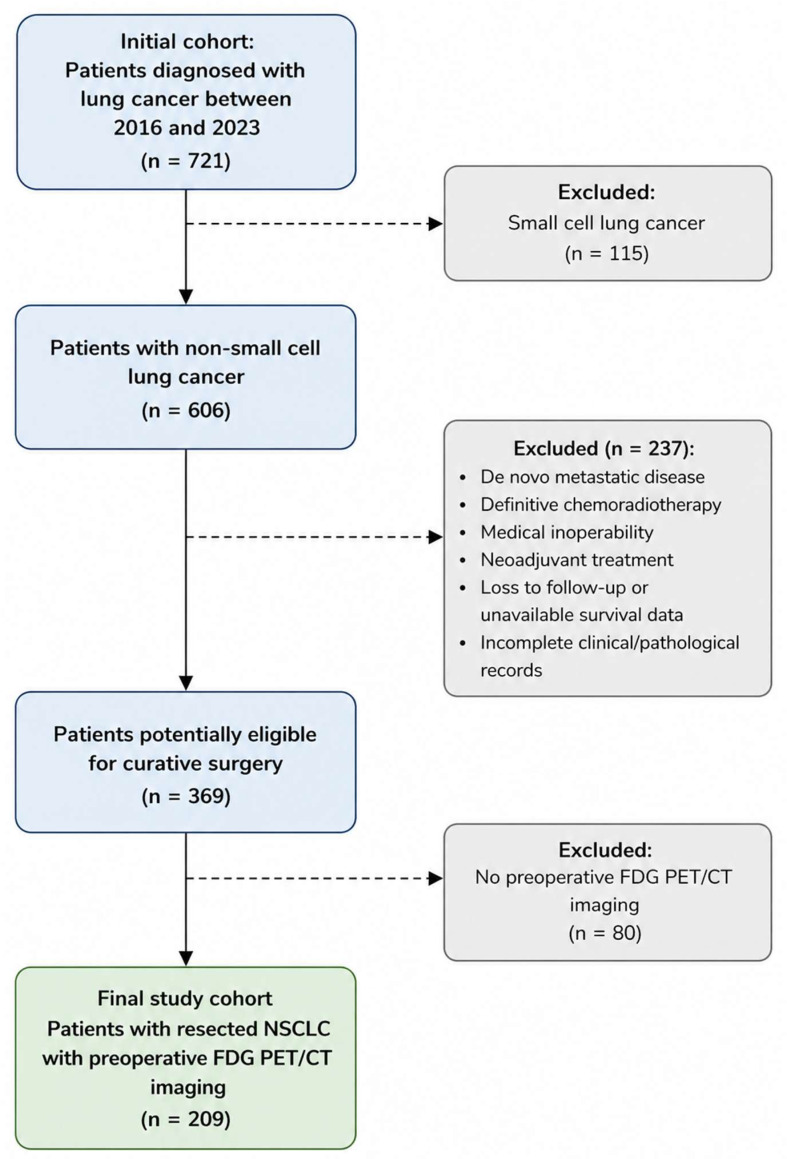
Flow chart of patient selection. Of 721 patients screened, those with small cell lung cancer, metastatic disease, neoadjuvant or non-surgical treatment, incomplete data, or missing preoperative FDG PET/CT were excluded. A total of 209 patients with resected NSCLC were included in the final analysis.

Patients were excluded if they had small cell lung cancer, de novo metastatic disease, definitive chemoradiotherapy without surgery, medical inoperability, neoadjuvant treatment before surgery, unavailable preoperative FDG PET/CT data, incomplete clinical or pathological records, or unavailable follow-up/survival data. From an initial cohort of 721 patients diagnosed with lung cancer, 115 patients with small cell lung cancer were excluded. Of the remaining patients, 237 were excluded because of metastatic disease, definitive chemoradiotherapy, medical inoperability, neoadjuvant treatment, loss to follow-up or unavailable survival data, or incomplete clinical/pathological records. A further 80 patients without preoperative FDG PET/CT were excluded, leaving 209 patients for the final analysis (Figure 1).

All patients were staged according to the eighth edition of the TNM classification. Demographic and clinical variables included age, sex, smoking status, Eastern Cooperative Oncology Group performance status, surgical procedure, and receipt of adjuvant therapy. Tumor-related variables included histologic subtype, pathological T stage, pathological TNM stage, primary tumor size, LVI, PNI, STAS, visceral pleural involvement, main bronchus involvement, extracapsular invasion, and surgical margin status.

The highest SUVmax of the primary tumor on preoperative FDG PET-CT was recorded for each patient. The prognostic performance of SUVmax for mortality was evaluated using ROC curve analysis, and the optimal cutoff was determined by the Youden index.

Patients were categorized into low-SUVmax (<11.14) and high-SUVmax (≥11.14) groups according to this cutoff.

The primary endpoint was OS, defined as the interval from surgery to death from any cause or last follow-up. The secondary endpoint was DFS, defined as the interval from surgery to first documented recurrence/progression or last disease-free follow-up. Deaths without documented recurrence were censored for DFS analysis.

Demographic and clinical data (age, sex, smoking status), tumor characteristics (histologic subtype, pathologic stage, invasion features), treatment details (surgery type, adjuvant therapy), and outcomes were collected.

The primary endpoint was overall survival (OS), defined as the time from surgery to death. Secondary endpoint was disease-free survival (DFS), defined as the time from surgery to first documented recurrence or progression. Survival was assessed by Kaplan–Meier analysis and compared using the log-rank test.

### Statistical Analysis

Continuous variables were summarized as mean ± standard deviation (SD) or median (minimum–maximum) according to data distribution, whereas categorical variables were presented as frequencies and percentages. Normality of continuous variables was assessed using visual methods (histograms and probability plots) and the Kolmogorov–Smirnov/Shapiro–Wilk test as appropriate.

Comparisons between the low-SUVmax and high-SUVmax groups were performed using the independent-samples *t*-test for normally distributed continuous variables and the Mann–Whitney U test for non-normally distributed variables. Categorical variables were compared using the Pearson chi-square test or Fisher’s exact test when appropriate.

Survival distributions (OS, DFS) were estimated using the Kaplan–Meier method and compared with the log-rank test.

Receiver operating characteristic (ROC) curve analysis was performed to evaluate the discriminatory ability of primary tumor SUVmax for mortality. The optimal SUVmax cutoff was determined using the Youden index, which identifies the point maximizing the sum of sensitivity and specificity. This approach was chosen because it provides an objective and reproducible threshold for dichotomizing a continuous biomarker in prognostic studies.

Univariate Cox proportional hazards regression analyses were first performed to identify variables potentially associated with OS and DFS. Variables with *p* < 0.10 in univariate analysis and variables considered clinically relevant based on previous literature were entered into the multivariable Cox regression models. Hazard ratios (HRs) and 95% confidence intervals (CIs) were reported.

The proportional hazards assumption for Cox regression was assessed using log-minus-log survival plots and Schoenfeld residual-based methods. Multicollinearity among covariates was evaluated before inclusion in the final multivariable models. Statistical analyses were performed using IBM SPSS Statistics version 25.0 (IBM Corp., Armonk, NY, USA, and a two-sided *p*-value < 0.05 was considered statistically significant.

## 3. Results

### 3.1. Patient and Tumor Characteristics

The study cohort (n = 209) had a median age of 62 years (range 41–82) and was 77.0% male. Most patients were heavy smokers. Tumor histology was adenocarcinoma in 92 patients (44.0%), squamous cell carcinoma in 90 (43.1%), and other types in 27 (12.9%). Pathologic stage distribution was 39.7% stage I (n = 83), 33.5% stage II (n = 70), and 26.8% stage III (n = 56). The median follow-up time was longer in the low-SUV group than the high-SUV group (*p* = 0.033) (Table 1).

### 3.2. Association of SUVmax with Clinicopathologic Variables

**Figure 2:**
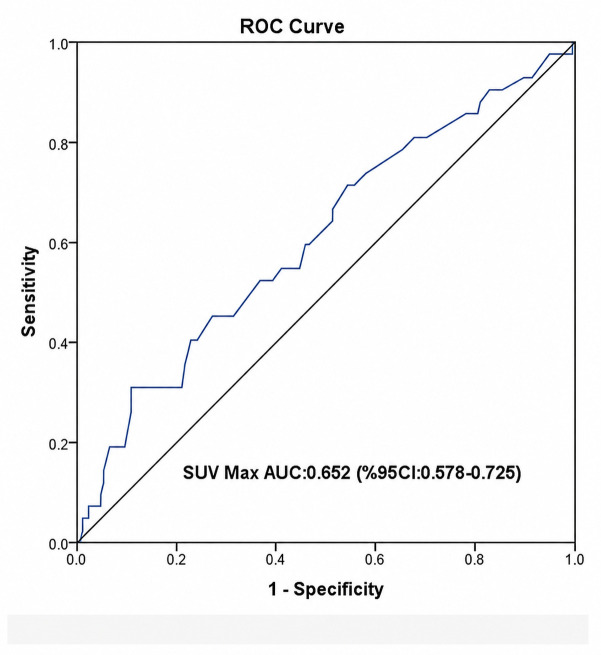
ROC curve analysis demonstrated that primary tumor SUVmax predicted mortality with an AUC of 0.652 (95% CI, 0.578–0.725). A cutoff value of 11.14 yielded 58.1% sensitivity and 57.8% specificity (*p* = 0.026).

Preoperative tumor SUVmax values ranged broadly (median 12.2, range 1.0–47.0). By ROC analysis, a cutoff of 11.14 was chosen (AUC = 0.652, 95% CI 0.578–0.725; *p* = 0.026) to best distinguish patients by mortality. We therefore compared patients with SUVmax < 11.14 (n = 113) vs. ≥ 11.14 (n = 96) (Figure 2). High-SUV tumors were significantly different in several aspects. Histologically, squamous cell carcinoma was more frequent in the high-SUV group (55.2% vs. 32.7%), whereas adenocarcinoma predominated in the low-SUV group (57.5% vs. 28.1%; *p* < 0.001). T stage and overall pathologic stage were significantly higher in the high-SUV group (*p* < 0.001); for example, 55.8% of low-SUV tumors were T1 versus only 17.7% of high-SUV tumors. Consequently, 54.0% of low-SUV patients were pathologic stage I compared to 22.9% of high-SUV patients (*p* < 0.001). High-SUV tumors were larger (mean diameter 4.57 cm vs. 2.80 cm; *p* < 0.001). Regarding treatment, more high-SUV patients underwent pneumonectomy (25% vs. 5.3%) and more received adjuvant chemotherapy (84.4% vs. 54.0%; *p* < 0.001). High SUVmax was also associated with aggressive pathologic features: extracapsular (soft tissue) invasion was more common (29.8% vs. 15.0%; *p* = 0.013), and perineural invasion occurred in 31.3% of high-SUV tumors versus 17.7% of low-SUV (*p* = 0.022). Surgical margin positivity was higher in the high-SUV group (10.4% vs. 2.7%; *p* = 0.021). In summary, tumors with SUVmax ≥ 11.14 were significantly more advanced and invasive than those below the cutoff (Table 1).

### 3.3. Survival Analysis

**Figure 3:**
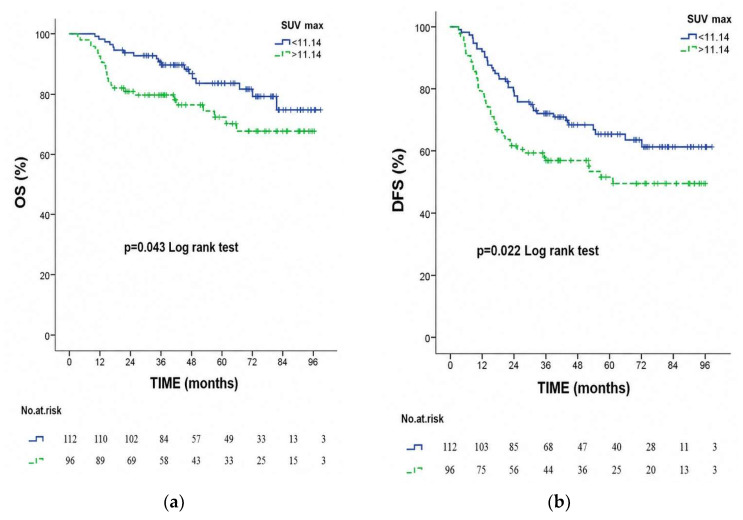
Kaplan–Meier survival curves stratified by primary tumor SUVmax. (**a**) Overall survival (OS) according to SUVmax (<11.14 vs. ≥11.14). Patients with higher SUVmax demonstrated significantly worse OS (log-rank *p* = 0.043). (**b**) Disease-free survival (DFS) according to SUVmax (<11.14 vs. ≥11.14). Higher SUVmax was associated with significantly shorter DFS (log-rank *p* = 0.022).

During follow-up, 43 patients (20.6%) died and 83 (39.7%) experienced recurrence or progression. The overall 2-year and 5-year OS rates were 88.0% and 78.8%, respectively. Patients with SUVmax < 11.14 had significantly better survival: their 2-year and 5-year OS rates exceeded those of the ≥11.14 group (log-rank *p* = 0.043) (Figure 3). Similarly, DFS was superior in the low-SUV group (2-year/5-year DFS 73.1%/63.3% for SUV < 11.14 vs. 65.5%/51.0% for SUV ≥ 11.14; *p* = 0.022) (Figure 3). These differences indicate that higher preoperative SUVmax is associated with reduced long-term survival and disease control on unadjusted analysis (Table 2 and Table 3).

In univariate Cox analysis, factors significantly associated with mortality included higher T stage, higher pathologic stage, receipt of adjuvant chemotherapy, extracapsular invasion, lymphovascular invasion, spread through air spaces (STAS), positive surgical margins, and SUVmax ≥ 11.14. In the multivariate Cox model for OS, only extracapsular invasion remained an independent predictor (HR = 3.60, 95% CI 1.41–9.17, *p* = 0.007). Preoperative SUVmax was not an independent predictor of OS: the HR for SUVmax ≥ 11.14 was 1.01 (*p* = 0.981) (Table 4).

For DFS, univariate analysis identified similar adverse factors (including high SUVmax). In multivariate analysis, only receipt of adjuvant chemotherapy was independently associated with progression risk (HR = 3.30, 95% CI 1.47–7.38, *p* = 0.004). Again, SUVmax ≥ 11.14 was not significant for DFS on multivariate analysis (HR = 1.15, *p* = 0.594). In summary, although high SUVmax stratified patients into groups with different unadjusted survival, it did not independently predict outcomes when other pathologic features were considered (Table 5).

## 4. Discussion

In this single-center cohort of patients with resected stage I-III NSCLC, high preoperative primary tumor SUVmax was associated with more aggressive clinicopathologic features and inferior OS and DFS in unadjusted analyses. However, SUVmax did not remain an independent predictor of OS or DFS after adjustment for established pathological risk factors. These findings suggest that SUVmax should not be interpreted as a standalone prognostic determinant after surgical pathology is available but rather as a non-invasive preoperative surrogate of aggressive disease biology.

This interpretation is clinically important. In our cohort, higher SUVmax was associated with adverse pathological features such as more advanced T stage, higher pathological stage, extracapsular invasion, PNI, surgical margin positivity, and a greater likelihood of relapse. Other established pathological factors, including STAS and LVI, also reflect aggressive tumor behavior and are known to influence recurrence risk. The key limitation is that most of these variables are identified only after resection. Therefore, they cannot reliably guide preoperative decision-making, surgical planning, or the selection of additional staging procedures. In contrast, SUVmax is available before surgery and may provide an early signal that the tumor has a higher-risk biological phenotype.

From a practical perspective, a high preoperative SUVmax may prompt clinicians to reassess whether the patient has been staged sufficiently before definitive surgery. This may include careful review of contrast-enhanced thoracic CT and PET/CT findings, consideration of invasive mediastinal staging with endobronchial ultrasound-guided sampling, or mediastinoscopy when nodal disease is suspected or when PET findings are equivocal, and discussion in a multidisciplinary tumor board. In selected patients, high metabolic activity may also support consideration of neoadjuvant systemic therapy with or without immunotherapy, chemoradiotherapy, or a more cautious approach to limited resection. Thus, SUVmax may be clinically useful not because it independently predicts outcome after all pathological variables are known, but because it can influence preoperative risk stratification while those variables remain unavailable.

Our results are consistent with previous studies showing that elevated FDG uptake is associated with worse outcomes after curative-intent treatment. A foundational meta-analysis by the European Lung Cancer Working Party reported that patients with high tumor SUVmax had roughly double the hazard of death compared to those with low uptake. More recent analyses focused on surgical cohorts have reinforced the link between elevated SUVmax and recurrence risk. For example, Kocaman et al. observed in a cohort of 314 pathologic stage I NSCLC patients that SUVmax > 5.2 was associated with significantly worse recurrence-free and overall survival and was an independent prognostic factor in multivariable analysis [7]. In another large retrospective curative-intent NSCLC cohort, Whi et al. showed that primary tumor SUVmax provided independent prognostic information for OS and DFS beyond the AJCC 9th edition staging system [10]. Likewise, an explainable machine-learning model in 643 resected NSCLC patients (Iguchi et al.) identified SUVmax as the single most important preoperative predictor of postoperative recurrence risk [11].

Interestingly, the relationship between SUVmax and recurrence in a model was nonlinear—the recurrence hazard rose sharply once SUVmax exceeded relatively low values (~2–5) [9]. This aligns with reports in early-stage disease that even modest FDG avidity portends higher metastatic potential. Chou et al., for instance, found that stage IA tumors with SUVmax > 4 had a dramatically increased hazard of relapse (on the order of 9-fold) compared to those with SUV ≤ 4 (*p* < 0.001) in their series [8]. Evidence from non-surgical local therapy settings (e.g., SBRT) also indicates that high SUVmax can reflect aggressive behavior, reinforcing its role as a general biologic marker rather than a surgery-specific phenomenon [12].

On the other hand, several studies—including our own—have noted that SUVmax may lose prognostic significance when analyzed alongside established pathological prognosticators. Tapias et al. (Mayo Clinic) recently examined 251 patients with resected clinical stage IA NSCLC and found that while a high SUVmax was associated with shorter time to recurrence on univariate analysis (HR ~1.6, *p* = 0.036), this association disappeared in multivariable modeling (adjusted HR ~1.5, *p* = 0.11). SUVmax was not an independent predictor of overall survival in that cohort [9]. Similarly, in a large-scale study of 516 lung cancer patients who underwent preoperative PET, Pini et al. reported that SUVmax correlated strongly with outcome on unadjusted analysis but had no independent prognostic value for progression-free survival after controlling for factors like tumor size and nodal status [13]. In their multivariate analysis, traditional factors such as pathologic stage and histology accounted for the prognostic information, and SUVmax did not significantly improve risk stratification [13]. The likely explanation is that high SUVmax is a surrogate for underlying tumor biology that is itself prognostic—larger tumors, nodal metastases, higher-grade histology, etc. Indeed, we observed that the high-SUV group in our study had significantly more advanced T stage, higher rates of nodal involvement, and more frequent extracapsular and perineural invasion than the low-SUV group. These aggressive pathologic features were the true drivers of recurrence and mortality risk, diminishing the apparent effect of SUVmax in multivariable models. In essence, once one knows the comprehensive pathology of the resected tumor, the preoperative SUVmax adds little additional prognostic information because it has been reflecting those same adverse features [13].

Notably, the optimal SUVmax threshold distinguishing high-risk vs. low-risk disease has varied widely across studies, underscoring the challenge of generalizing a single cutoff. We derived a cutoff of 11.14 by ROC analysis for all-cause mortality in our mixed-stage population, which is considerably higher than the cutoffs around 4–6 often reported in early-stage cohorts [11,13]. This discrepancy likely arises because our study included a substantial proportion of stage II–III tumors, which tend to have higher FDG uptake; a much higher threshold was needed to differentiate outcomes in this heterogeneous group. By contrast, in pure stage I cohorts, very low SUV cutpoints (~3–5) can segregate indolent vs. aggressive behavior [7,14]. More broadly, a recent review of the literature found SUV cutoffs used for prognostication ranging anywhere from 2.5 up to 15 [13]. These thresholds were defined by various methods (ROC-derived “optimal” cutpoints, median split, arbitrary values, etc.), contributing to inter-study heterogeneity [13]. Even when focusing only on the most robust studies, there remains neither consensus optimal SUV threshold for risk stratification nor definitive proof of an independent prognostic effect of SUVmax across all settings. This variability cautions against uncritical adoption of any single SUV cutoff in clinical practice. Our chosen cutpoint of 11.14 provided clear stratification in our dataset, but it may not be applicable to different patient populations or PET scanner protocols. In fact, the relatively high threshold in our study likely reduced sensitivity—consistent with our ROC AUC of only ~0.65—meaning many patients with moderately elevated SUV (e.g., 6–10) still experienced events despite being below the cut. Thus, rather than a universal dichotomous threshold, a more nuanced approach (e.g., treating SUVmax as a continuous risk factor or integrating it into multivariable risk models) might be preferable going forward [13].

The clinical implications of a high preoperative SUVmax in resectable NSCLC warrant consideration, even if SUVmax is not an independent predictor by itself. First, FDG-avid tumors could influence surgical decision-making. There is evidence that high SUV tumors are more likely to harbor occult nodal metastases [9] and have worse local control with limited resection. Kocaman et al. observed no difference in recurrence-free survival between lobectomy versus sublobar resection for stage I patients with low-SUV tumors, but among patients with SUVmax > 5.2, those who underwent sublobar resection had significantly higher recurrence rates than those who had lobectomy (5-year RFS ~53% vs. 77%) [7]. Similarly, Tapias et al. suggested that SUVmax should be considered a risk factor when evaluating patients for sublobar surgery in stage I disease [9]. These data suggest that an FDG-hot lesion, even if small, might merit more aggressive surgical management (anatomic lobectomy and thorough lymph node dissection) to mitigate recurrence risk. Second, SUVmax may help identify patients who would benefit from additional therapy after surgery. Our multivariate analysis indicated that adjuvant chemotherapy was associated with improved DFS, and this appeared to particularly benefit those with high-SUV tumors (since a larger proportion of the high-SUV group received adjuvant treatment). In line with this, a recent large study of 928 patients by Huang et al. found that within pathologic stage I lung adenocarcinoma, the subgroup with SUVmax ≥ 5.0 derived a significant survival benefit from adjuvant chemotherapy, whereas those with SUVmax < 5.0 saw no benefit [15]. In other words, PET metabolic activity helped pinpoint “high-risk” stage I patients who were most likely to gain from adjuvant therapy. Although prospective trials have not yet incorporated SUV-based criteria, these retrospective findings hint that tailoring postoperative management based on preoperative SUVmax (in conjunction with standard pathologic risk factors) could improve outcomes. At the very least, a high SUVmax tumor should prompt vigilant surveillance after resection, since these patients are at an elevated risk for recurrence even when pathology is favorable. In the context of current practice, clinicians might reasonably use a high PET uptake value as an additional piece of the risk assessment puzzle—for example, to justify closer follow-up imaging or to discuss the potential benefits of adjuvant systemic therapy in borderline cases.

Preoperative risk stratification in resectable NSCLC should therefore be multimodal rather than PET-centered. FDG PET/CT contributes important metabolic and whole-body staging information, but it should be interpreted together with anatomical imaging, invasive nodal assessment when indicated, pulmonary and cardiac functional evaluation, and careful clinical assessment. Selected use of cardiovascular imaging may also be relevant. Cardiac metastases or malignant cardiac masses are uncommon in patients being evaluated for resectable NSCLC, but when suspected they can substantially alter prognosis and management. Echocardiography is an accessible non-invasive tool that can help detect and characterize cardiac masses in patients with suggestive symptoms, pericardial effusion, equivocal imaging findings, arrhythmia, hemodynamic compromise, or suspected intracardiac involvement. Paolisso et al. reported that echocardiographic features can help predict the malignant nature of cardiac masses, supporting echocardiography as part of a multimodality diagnostic pathway in selected oncology patients [16].

Future directions: Further research is warranted to refine the role of metabolic imaging in prognostication and to integrate it with emerging biomarkers. Prospective validation of SUVmax-based risk stratification in resectable NSCLC should be pursued—for example, incorporating SUV thresholds into trial designs or staging systems to see if they improve risk prediction above TNM staging. It may also be efficient to move beyond SUVmax alone. Recent studies have shown that volumetric PET parameters like metabolic tumor volume (MTV) and total lesion glycolysis (TLG) can provide prognostic information independent of SUVmax [14,17,18,19], and in our data MTV was moderately correlated with SUVmax (though we did not formally analyze MTV prognostically). Combining SUVmax with other quantitative metrics and pathological features could yield composite risk models with superior discrimination. Zheng et al. demonstrated this principle in stage I lung adenocarcinoma by integrating SUVmax with tumor grade and CT imaging features: patients classified as “high-risk” by this multi-factor model (SUVmax > 3.9, MTV > 5.4 mL, grade 3, or presence of cavity) had a 5-year RFS of ~61% vs. 94% in the low-risk group (HR 6.0) [14]. Such multi-parameter stratification could guide personalized therapy intensification for those most at risk. Another promising avenue is PET–genomic integration or “radiogenomics.” Transcriptomic and mutational profiling of tumors might be combined with metabolic imaging to identify correlations (for example, SUVmax with gene expression signatures of tumor hypoxia, proliferation, or immune evasion). Early work has linked high FDG uptake with upregulation of glucose transporter type 1 (GLUT-1) and glycolytic enzymes, as well as with high programmed death-ligand 1 (PD-L1) expression, suggesting that hypermetabolic tumors have distinct molecular phenotypes. Going forward, linking PET data with genomic data (e.g., constructing prognostic nomograms that include SUV and relevant gene mutations or immune markers) could improve risk assessment and even uncover novel therapeutic targets for biologically aggressive, FDG-avid cancers. Lastly, as new systemic therapies emerge in the adjuvant setting (including immunotherapies and targeted agents), it will be worth exploring whether PET metrics can help select patients who stand to benefit most from these treatments. In summary, while SUVmax alone may not ultimately serve as a formal independent prognostic factor when all is said and done, it remains a valuable piece of the puzzle. Continued research should aim to leverage the information it provides—in concert with pathologic and molecular data—to enhance our ability to stratify early-stage NSCLC patients by risk and to tailor their management accordingly.

## 5. Conclusions

In patients undergoing resection for NSCLC, a high preoperative tumor SUVmax (≥11.14) was associated with more advanced pathologic features and worse survival rates. However, SUVmax was not an independent prognostic factor after accounting for tumor invasiveness and other variables. Extracapsular invasion and receipt of adjuvant chemotherapy emerged as the strongest predictors of outcome. Preoperative SUVmax may help flag high-risk patients but should be integrated with comprehensive clinicopathologic assessment. Prospective studies are needed to confirm an optimal SUVmax cutoff and to determine how PET-derived metrics can best guide treatment planning.

## Figures and Tables

**Table 1:** Comparison of sociodemographic and clinical variables based on SUVmax values.

		SUVmax		
Variables	Total N = 209	<11.14 N = 113	>11.14 N = 96	*p*
Age				
Mean ± SD	61.94 ± 8.06	62.29 ± 8.28	61.52 ± 7.82	0.492 c
Median (min–max)	62.0 (41–82)			
<60 years	77 (36.8)	40 (35.4)	37 (38.5)	0.639 a
>60 years	132 (63.2)	73 (64.6)	59 (61.5)	
Gender				
Female	48 (23.0)	28 (24.8)	20 (20.8)	0.499 a
Male	161 (77.0)	85 (75.2)	76 (79.2)	
Histology				
Adeno	92 (44.0)	65 (57.5)	27 (28.1)	**<0.001 a**
Squamous	90 (43.1)	37 (32.7)	53 (55.2)	
Other	27 (12.9)	11 (9.7)	16 (16.7)	
T stage				
T1	80 (38.3)	63 (55.8)	17 (17.7)	**<0.001 a**
T2	57 (27.3)	22 (19.5)	35 (36.5)	
T3	45 (21.5)	19 (16.8)	26 (27.1)	
T4	27 (12.9)	9 (8)	18 (18.8)	
Pathological stage (TNM)				
Stage-1	83 (39.7)	61 (54)	22 (22.9)	**<0.001 a**
Stage-2	70 (33.5)	28 (24.8)	42 (43.8)	
Stage-3	56 (26.8)	24 (21.2)	32 (33.3)	
Primary tumor size				
Mean ± SD	3.61 ± 1.97	2.80 ± 1.74	4.57 ± 1.80	**<0.001 c**
Median (min–max)	3.00 (0.60–10.50)			
Smoking				
No	27 (13.2)	18 (16.5)	9 (9.5)	0.139 a
Yes	177 (86.8)	91 (83.5)	86 (90.5)	
ECOG PS				
0	121 (58.5)	59 (52.7)	62 (65.3)	0.241 b
1	77 (37.2)	47 (42)	30 (31.6)	
2	7 (3.4)	5 (4.5)	2 (2.1)	
3	2 (1.0)	1 (0.9)	1 (1.1)	
PD-L1 percentage				
Mean ± SD	14.72 ± 27.56	4.90 ± 7.07	27.00 ± 38.34	0.091 c
Median (min–max)	1.5 (0.0–90.0)			
Type of surgery				
Wedge resection	18 (8.6)	16 (14.2)	2 (2.1)	**<0.001 b**
Lobectomy	155 (74.2)	86 (76.1)	69 (71.9)	
Pneumonectomy	30 (14.4)	6 (5.3)	24 (25)	
Bilobectomy	6 (2.9)	5 (4.4)	1 (1)	
Adjuvant Chemotherapy				
No	67 (32.1)	52 (46)	15 (15.6)	**<0.001 a**
Yes	142 (67.9)	61 (54)	81 (84.4)	
Extracapsular invasion				
Negative	150 (78.5)	91 (85)	59 (70.2)	**0.013 a**
Positive	41 (21.5)	16 (15)	25 (29.8)	
Main bronchus involvement				
Negative	166 (83.8)	96 (87.3)	70 (79.5)	0.142 a
Positive	32 (16.2)	14 (12.7)	18 (20.5)	
LVI				
Negative	135 (64.6)	79 (69.9)	56 (58.3)	0.081 a
Positive	74 (35.4)	34 (30.1)	40 (41.7)	
PNI				
Negative	159 (76.1)	93 (82.3)	66 (68.8)	**0.022 a**
Positive	50 (23.9)	20 (17.7)	30 (31.3)	
STAS				
Negative	138 (66.3)	78 (69.6)	60 (62.5)	0.277 a
Positive	70 (33.7)	34 (30.4)	36 (37.5)	
Surgical margin				
Negative	196 (93.8)	110 (97.3)	86 (89.6)	**0.021 a**
Positive	13 (6.2)	3 (2.7)	10 (10.4)	
Visceral pleural involvement				
Negative	142 (67.9)	77 (68.1)	65 (67.7)	0.947 a
Positive	67 (32.1)	36 (31.9)	31 (32.3)	
Relapse				
No	126 (60.3)	75 (66.4)	51 (53.1)	0.051 a
Yes	83 (39.7)	38 (33.6)	45 (46.9)	
Mortality				
Alive	166 (79.4)	95 (84.1)	71 (74)	0.072 a
Exitus	43 (20.6)	18 (15.9)	25 (26)	
Follow-up period (months)				
Mean ± SD	51.48 ± 25.69	55.00 ± 23.28	47.38 ± 27.81	**0.033 c**
Median (min–max)	46.58 (3.13–98.23)			

**a: Pearson Chi Square test, b: Fisher’s Exact test, c: Independent *t*-test, *p* < 0.05 statistically significant.** SUVmax: Maximum Standardized Uptake Value, TNM: Tumor, Node, and Metastasis ECOG PS: Eastern Cooperative Oncology Group Performance Status, PD-L1: Programmed Death-Ligand 1, LVI: Lymphovascular Invasion, PNI: Perineural Invasion, STAS: Spread Through Air Spaces.

**Table 2:** Overall survival comparisons for patients.

Variables	2 Years %	5 Years %	*p*
All population	88.0	78.8	
Age			
<60	88.0	80.9	0.615
>60	84.4	76.1	
Gender			
Female	91.6	80.4	0.105
Male	84.8	70.4	
Histology			
Adeno	86.8	71.1	0.778
Squamous	82.2	70.6	
Other	94.2	82.5	
T stage			
T1	92.9	81.9	0.146
T2	85.5	72.0	
T3	80.0	65.2	
T4	74.1	64.5	
Pathological stage (TNM)			
Stage-1	94.0	88.3	**0.011**
Stage-2	82.6	75.7	
Stage-3	85.6	67.6	
Smoking			
No	88.0	72.4	0.507
Yes	86.0	73.3	
ECOG PS			
0	89.1	77.9	0.281
1	82.2	65.8	
2	64.6	65.5	
3	50.0	-	
Type of surgery			
Wedge resection	90.1	81.0	0.057
Lobectomy	85.5	72.0	
Pneumonectomy	79.4	61.0	
Bilobectomy	-	66.3	
Adjuvant chemotherapy			
No	93.9	86.3	**0.035**
Yes	85.2	75.1	
Extracapsular invasion			
Negative	89.3	83.2	**0.016**
Positive	85.4	62.4	
LVI			
Negative	91.8	82.0	**0.007**
Positive	81.1	70.1	
PNI			
Negative	87.9	75.5	0.279
Positive	78.8	65.1	
STAS			
Negative	92.0	89.0	**<0.001**
Positive	80.0	58.4	
Main bronchus involvement			
Negative	83.9	75.0	0.054
Positive	80.1	69.6	
Surgical margin			
Negative	89.7	81.7	**<0.001**
Positive	61.5	30.8	
Visceral pleural involvement			
Negative	86.4	75.0	0.486
Positive	84.0	70.9	
SUVmax			
<11.14	93.7	83.9	**0.043**
≥11.14	81.2	72.8	

**Kaplan–Meier, Log rank test, *p* < 0.05 statistically significant.** TNM: Tumor, Node, and Metastasis, ECOG PS: Eastern Cooperative Oncology Group Performance Status, LVI: Lymphovascular Invasion, PNI: Perineural Invasion, STAS: Spread Through Air Spaces, SUVmax: Maximum Standardized Uptake Value.

**Table 3:** Disease-free survival comparisons for patients.

Variables	2 Years %	5 Years %	*p*
All population	70.6	58.8	
Age			
<60	73.1	63.3	0.733
>60	65.5	51.0	
Gender			
Female	78.0	63.0	0.305
Male	65.0	53.2	
Histology			
Adeno	67.1	50.3	0.430
Squamous	65.9	54.3	
Other	84.0	76.0	
T stage			
T1	82.5	69.6	0.007
T2	71.8	54.7	
T3	58.4	55.9	
T4	51.6	39.7	
Pathological stage (TNM)			
Stage-1	81.9	64.9	0.010
Stage-2	71.0	62.1	
Stage-3	53.2	45.6	
Smoking			
No	67.7	49.4	0.465
Yes	68.4	55.0	
ECOG PS			
0	73.9	56.6	0.805
1	62.9	52.2	
2	45.4	35.1	
3	50.0	-	
Type of surgery			
Wedge resection	76.3	71.0	0.096
Lobectomy	71.0	57.3	
Pneumonectomy	55.8	41.5	
Bilobectomy	54.0	36.0	
Adjuvant chemotherapy			
No	84.8	67.4	0.007
Yes	63.9	54.4	
Extracapsular invasion			
Negative	74.6	62.9	0.038
Positive	58.0	45.7	
LVI			
Negative	78.2	64.5	0.006
Positive	56.7	48.3	
PNI			
Negative	70.3	55.4	0.112
Positive	55.8	49.2	
STAS			
Negative	80.4	71.1	<0.001
Positive	51.1	33.5	
Main bronchus involvement			
Negative	68.4	55.0	0.335
Positive	65.0	45.0	
Surgical margin			
Negative	72.7	60.8	0.001
Positive	38.5	-	
Visceral pleural involvement			
Negative	74.6	62.7	0.148
Positive	61.8	50.2	
SUVmax			
<11.14	78.4	65.2	
≥11.14	61.4	0.022	

TNM: Tumor, Node, and Metastasis, ECOG PS: Eastern Cooperative Oncology Group Performance Status, LVI: Lymphovascular Invasion, PNI: Perineural Invasion, STAS: Spread Through Air Spaces, SUVmax: Maximum Standardized Uptake Value.

**Table 4:** Multivariate Cox regression results on the mortality risk of various clinical variables.

Variables	HR (%95 CI)	*p*
TNM staging		0.136
Stage-1	ref	
Stage-2	2.53 (0.92–6.97)	0.072
Stage-3	1.52 (0.51–4.55)	0.451
Adjuvant chemotherapy		
No	ref	0.919
Yes	0.94 (0.34–2.61)	
Extracapsular invasion		
Negative	ref	**0.007**
Positive	3.60 (1.41–9.17)	
LVI		
Negative	ref	0.352
Positive	1.15 (0.70–2.70)	
STAS		
Negative	ref	0.500
Positive	1.02 (0.44–3.28)	
Surgical margin		
Negative	ref	0.700
Positive	1.43 (0.10–4.32)	
SUVmax		
<11.14	ref	0.981
≥11.14	1.01 (0.50–2.03)	

**−2 Log Likelihood:360.398, *p* < 0.001.** TNM: Tumor, Node, and Metastasis, LVI: Lymphovascular Invasion, STAS: Spread Through Air Spaces, SUVmax: Maximum Standardized Uptake Value.

**Table 5:** Multivariate Cox regression results on the effect of various clinical variables on the risk of recurrence.

Variables	HR (%95 CI)	*p*
T stage		0.123
T1	ref	
T2	2.80 (1.65–5.33)	0.059
T3	1.60 (0.98–3.60)	0.930
T4	1.39 (0.60–5.80)	0.470
TNM staging		0.932
Stage-1	ref	
Stage-2	0.92 (0.44–1.92)	0.843
Stage-3	0.84 (0.35–2.02)	0.708
Adjuvant chemotherapy		
No	ref	**0.004**
Yes	3.30 (1.47–7.38)	
Extracapsular invasion		
Negative	ref	0.059
Positive	1.46 (0.33–7.80)	
LVI		
Negative	ref	0.138
Positive	1.11 (0.80–2.59)	
STAS		
Negative	ref	0.125
Positive	1.30 (0.10–2.60)	
Surgical margin		
Negative	ref	0.099
Positive	1.45 (0.20–2.90)	
SUVmax		
<11.14	ref	0.594
≥11.14	1.15 (0.68–1.93)	

**−2 Log Likelihood: 707.08, *p* < 0.001.** TNM: Tumor, Node, and Metastasis, LVI: Lymphovascular Invasion, STAS: Spread Through Air Spaces, SUVmax: Maximum Standardized Uptake Value.

## Data Availability

The original contributions presented in this study are included in the article. Further inquiries can be directed to the corresponding author(s).

## Abbreviations

The following abbreviations are used in this manuscript:

FDG	Fluorodeoxyglucose
SUVmax	Maximum Standardized Uptake Value
OS	Overall Survival
DFS	Disease-Free Survival
ROC	Receiver Operating Characteristic
AUC	Area Under the Curve
CI	Confidence Interval
HR	Hazard Ratio
TNM	Tumour, Node, Metastasis
ECOG PS	Eastern Cooperative Oncology Group Performance Status
PD-L1	Programmed Death-Ligand 1
LVI	Lymphovascular Invasion
PNI	Perineural Invasion
STAS	Spread Through Air Spaces
MTV	Metabolic Tumor Volume
TLG	Total Lesion Glycolysis
GLUT-1	Glucose transporter type 1

## References

[1] Qiu X., Liang H., Zhong W., Zhao J., Chen M., Zhu Z., Xu Y., Wang M. (2021). Prognostic impact of maximum standardized uptake value on (18) f-fdg pet/ct imaging of the primary lung lesion on survival in advanced non-small cell lung cancer: A retrospective study. Thorac. Cancer.

[2] Liu J., Dong M., Sun X., Li W., Xing L., Yu J. (2016). Prognostic value of 18f-fdg pet/ct in surgical non-small cell lung cancer: A meta-analysis. PLoS ONE.

[3] Berghmans T., Dusart M., Paesmans M., Hossein-Foucher C., Buvat I., Castaigne C., Scherpereel A., Mascaux C., Moreau M., Roelandts M. (2008). Primary tumor standardized uptake value (suvmax) measured on fluorodeoxyglucose positron emission tomography (fdg-pet) is of prognostic value for survival in non-small cell lung cancer (nsclc): A systematic review and meta-analysis (ma) by the european lung cancer working party for the iaslc lung cancer staging project. J. Thorac. Oncol..

[4] Paesmans M., Garcia C., Wong C.Y., Patz E.F., Komaki R., Eschmann S., Govindan R., Vansteenkiste J., Meert A.P., de Jong W.K. (2015). Primary tumour standardised uptake value is prognostic in nonsmall cell lung cancer: A multivariate pooled analysis of individual data. Eur. Respir. J..

[5] Blumenthaler A.N., Hofstetter W.L., Mehran R.J., Rajaram R., Rice D.C., Roth J.A., Sepesi B., Swisher S.G., Vaporciyan A.A., Walsh G.L. (2022). Preoperative maximum standardized uptake value associated with recurrence risk in early lung cancer. Ann. Thorac. Surg..

[6] Um S.W., Kim H., Koh W.J., Suh G.Y., Chung M.P., Kwon O.J., Choi J.Y., Han J., Lee K.S., Kim J. (2009). Prognostic value of 18f-fdg uptake on positron emission tomography in patients with pathologic stage i non-small cell lung cancer. J. Thorac. Oncol..

[7] Kocaman G., Ibrahımov F., Kahya Y., Araz M., Elhan A.H., Enön S. (2025). Suvmax of the lesion should be considered in the treatment plan for stage i non-small cell lung cancer. Ann. Nucl. Med..

[8] Chou H.P., Lin K.H., Huang H.K., Lin L.F., Chen Y.Y., Wu T.H., Lee S.C., Chang H., Huang T.W. (2021). Prognostic value of positron emission tomography in resected stage ia non-small cell lung cancer. Eur. Radiol..

[9] Tapias L.F., Shen R., Cassivi S.D., Reisenauer J.S., Lunn B.W., Lechtenberg B.J., Nichols F.C., Wigle D.A., Blackmon S.H. (2024). Impact of fdg pet standardized uptake value in resected clinical stage ia non-small cell lung cancer. Ann. Thorac. Surg..

[10] Whi W., Lee H., Um S.W., Kim H.K., Pyo H.R., Ahn M.J., Choi J.Y. (2025). Prognostic value of [(18)f]fluorodeoxyglucose pet/ct in the new staging system for non-small cell lung cancer. Eur. Radiol..

[11] Iguchi T., Kojima K., Hayashi D., Tokunaga T., Okishio K., Yoon H. (2025). Preoperative maximum standardized uptake value emphasized in explainable machine learning model for predicting the risk of recurrence in resected non-small cell lung cancer. JCO Clin. Cancer Inform..

[12] Horne Z.D., Clump D.A., Vargo J.A., Shah S., Beriwal S., Burton S.A., Quinn A.E., Schuchert M.J., Landreneau R.J., Christie N.A. (2014). Pretreatment suvmax predicts progression-free survival in early-stage non-small cell lung cancer treated with stereotactic body radiation therapy. Radiat. Oncol..

[13] Pini C., Kirienko M., Gelardi F., Bossi P., Rahal D., Toschi L., Ninatti G., Rodari M., Marulli G., Antunovic L. (2024). Challenging the significance of suv-based parameters in a large-scale retrospective study on lung lesions. Cancer Imaging.

[14] Zheng X., Lin J., Xie J., Jiang J., Lan J., Ji X., Tang K., Zheng X., Liu J. (2023). Evaluation of recurrence risk for patients with stage i invasive lung adenocarcinoma manifesting as solid nodules based on (18)f-fdg pet/ct, imaging signs, and clinicopathological features. EJNMMI Res..

[15] Huang M., Liu B., Li X., Li N., Yang X., Wang Y., Zhang S., Lu F., Li S., Yan S. (2024). Beneficial implications of adjuvant chemotherapy for stage ib lung adenocarcinoma exhibiting elevated suvmax in fdg-pet/ct: A retrospective study from a single center. Front. Oncol..

[16] Paolisso P., Foà A., Bergamaschi L., Graziosi M., Rinaldi A., Magnani I., Angeli F., Stefanizzi A., Armillotta M., Sansonetti A. (2023). Echocardiographic Markers in the Diagnosis of Cardiac Masses. J. Am. Soc. Echocardiogr..

[17] Harmon S., Seder C.W., Chen S., Traynor A., Jeraj R., Blasberg J.D. (2019). Quantitative fdg pet/ct may help risk-stratify early-stage non-small cell lung cancer patients at risk for recurrence following anatomic resection. J. Thorac. Dis..

[18] Melloni G., Gajate A.M., Sestini S., Gallivanone F., Bandiera A., Landoni C., Muriana P., Gianolli L., Zannini P. (2013). New positron emission tomography derived parameters as predictive factors for recurrence in resected stage i non-small cell lung cancer. Eur. J. Surg. Oncol..

[19] Tosi D., Pieropan S., Cattoni M., Bonitta G., Franzi S., Mendogni P., Imperatori A., Rotolo N., Castellani M., Cuzzocrea M. (2021). Prognostic value of 18f-fdg pet/ct metabolic parameters in surgically treated stage i lung adenocarcinoma patients. Clin. Nucl. Med..

